# Prevalence of Allergic Sensitization in Childhood Asthma

**DOI:** 10.7759/cureus.15311

**Published:** 2021-05-29

**Authors:** Bijay Kumar Meher, Deepti D Pradhan, Jatadhari Mahar, Sanjay K Sahu

**Affiliations:** 1 Pediatrics, Bhima Bhoi Medical College and Hospital, Balangir, IND; 2 Pediatrics, Kalinga Institute of Medical Sciences, Bhubaneswar, IND; 3 Pediatrics, Srirama Chandra Bhanja (SCB) Medical College and Hospital, Cuttack, IND

**Keywords:** asthma, allergic rhinitis, immunoglobulin e, skin prick test, recurrent wheezing

## Abstract

Introduction

The allergic sensitization in childhood asthma is common and the prevalence varies in different geographical locations. The data on allergen sensitization to guide clinicians on allergy avoidance is limited.

Method

This prospective observational study was conducted between October 2019 and April 2020 on children aged two to 14 years attending an outpatient clinic. Those with recurrent wheezing or physician-diagnosed asthma were interviewed; eosinophil count, total serum immunoglobulin E (IgE) was measured; and skin prick test (SPT) was done using standardized reagents. Wheal size of ≥3mm was considered positive.

Results

A total of 80 children were enrolled. The mean age was 71.15 ± 33.52 months (M:F ratio =1.96:1). Allergic rhinitis, conjunctivitis, and dermatosis were seen in 76 (95.0%), 33 (41.3%), and 22 (27.5%) cases, respectively. The mean absolute eosinophil count was 576 ± 427per cmm. The mean total IgE was 800.9 ± 883.2IU/ml. Seasonal and diurnal variations were found in 34 (42.5%) and 79 (98.8%) cases. Out of 1753 skin pricks using 27 reagents, 355 (20.25%) were positive. Increasing age was significantly associated with increasing SPT positivity (P = 0.0001). The most common sensitive aeroallergens were Kentucky bluegrass (25%), Dermatophagoides pteronyssinus (22.5%), Dermatophagoides farinae (21.3%), Timothy grass, and Alternaria alternans (20% each). The most common sensitive food allergens were spinach (25%), banana (22.5%), carp (20%), shrimp and hen’s egg (18.8% each), and cow’s milk (17.5%).

Conclusion

Increasing age was associated with increasing SPT positivity in childhood asthma. The most common sensitive aeroallergens were Kentucky bluegrass and Dermatophagoides pteronyssinus; spinach and banana were the most common food allergen.

## Introduction

Asthma is a heterogeneous disease, defined by the recurrent episodes of wheeze, shortness of breath, chest tightness, and cough that vary over time and intensity, together with variable expiratory airflow limitation [1]. Its prevalence is around 5-10% worldwide [2,3]. The diagnosis is based on characteristic symptom patterns, i.e., ≥3 episodes of wheezing over a 12-month period [4]. Atopy, a genetic predisposition to produce an excess amount of immunoglobulin E (IgE) antibodies when exposed to allergens is nearly a universal finding in children with asthma [5,6]. Allergic sensitization is a hypersensitivity reaction to a particular antigen, which can be assessed by in-vivo demonstration of bound IgE and mast cell degranulation by skin prick testing (SPT) or in-vitro tests to detect the production of free IgE to a specific allergen (sIgE) [7]. Allergic sensitization using SPT is not a routine practice and sensitization data on childhood asthma in India are scarce [8]. The present study was designed to find out the prevalence of atopy and allergy in childhood asthma by estimation of serum total IgE assay and to find out the spectrum of allergic sensitization to commonly selected aeroallergen and food allergen using SPT.

## Materials and methods

This was an observational study carried out in Kid's first outpatient clinic, Cuttack, an eastern city of Odisha state from October 2019 to April 2020. Approval was obtained from the institutional ethical committee, Balangir. Informed consent was obtained from the parents for enrolling children in this study. Children of age two to 14 years diagnosed with asthma (Global Initiative for Asthma {GINA} guidelines for diagnostic criteria of asthma in children) or have a history of recurrent (≥3 in the last 12 months) episodes of respiratory symptoms, such as wheezing, shortness of breath, chest tightness, and cough were included in the study. Seriously ill children requiring hospitalization and children with features suggestive of alternative diagnosis were excluded [9]. A sample size of 80 was calculated with a power of 0.81 for a 5% level of significance.

Demographic data like age, sex, socioeconomic status, anthropometric details, associated features of allergic rhinitis (AR), allergic conjunctivitis (AC), and allergic dermatosis (AD) were observed. AR was diagnosed with a history of nasal congestion, rhinorrhea, and itching often accompanied by sneezing. AC was diagnosed with ocular itching, tearing with injected or swollen conjunctiva. AD was diagnosed on the basis of the presence of pruritus, eczematous dermatitis with a chronic or relapsing course. A detailed history regarding seasonal variation, diurnal variation, location of symptoms (indoor/outdoor), and possible triggers was collected. Seasonal variation is the variation in a time series within a year that is repeated more or less regularly. Diurnal variation refers to the presence of symptoms that occur at a particular time of the day (e.g., during sleep) or even when symptoms occur throughout the day, worse at some time (e.g., night). Serum total IgE and blood eosinophil count were measured. IgE level was compared with the age-specific reference range as described by Barbee et al. and Kjellman et al. [10,11]. The patients' management was done as per the GINA guidelines [1].

Skin prick test

SPT was conducted after control of acute phase using Allergo SPT Kit (Merck Pharma, USA) with the available reagents selecting common allergens from different groups (dust mite, mold, grass and weed pollen, animal epithelia, foods like cow's milk, hen's egg, wheat, peanut, meat, seafood, fruits, and vegetables). In order to ascertain individual reactivity, control tests with buffered saline (negative control) and histamine (positive control) were carried out. 

One week before performing SPT, oral drugs including antihistamines, steroids, or any other drugs likely to affect SPT were discontinued but inhaled drugs were continued. But the cases, in those who were administered long-acting antihistamines, SPT was performed after four weeks of discontinuation of the drug. Twenty-seven different types of standardized allergens were selected, which included dust mite (two types), mold (four types), grass pollen (four types), weed pollen (three types), animal epithelia (one type), and food antigen (13 types). SPT was conducted by putting a drop of antigen on the healthy skin over volar aspects of both forearms (5cm away from the wrist and 3cm from antecubital fossa) with a dropper pipette at a gap of approximately 4cm between the drops. A shallow prick was made using the tip of a specially designed "lancet" through the drop of the test solution at 45° to 60° angle to the skin. The lancet was lifted slightly so that a small amount of test solution can penetrate the skin underneath the tip. Reading was interpreted after a lapse of 15-20 minutes. Skin reactivity was assessed by calculating the mean diameter against a scale provided with the kit. To establish test validity, positive control needed to be at least 3mm more than the negative control. Any reaction with normal saline more than 3mm was considered invalid. A positive result to a specific antigen was indicated by a wheal diameter measuring ≥3mm. Wheal diameter <3mm or less than negative control was taken as normal. 

Sample size

The chi-square test of association and the Mann-Whitney test are used in this analysis. The sample size used here was 80. For the chi-square test, this sample size achieved a power of 0.81 for 5% level of significance considering a medium effect size of 0.32 and 1 degree of freedom. This was computed using posthoc computed achieved power analysis using G. Power 3.2 software [12].

Statistical methods

The analysis was carried out using IBM SPSS Statistics 24.0 software (New York, USA: IBM Corp.). Descriptive statistics were calculated. The P-value <0.05 was considered statistically significant. For categorical variables, the test of independence was studied using cross-tabulation procedure and chi-square test of association. Frequency procedure was used to find out the distribution of demographic characteristics of the sample and allergic sensitization in the skin prick test. For scale variable comparison of two distributions was done using mean and standard deviation, median and interquartile range (IQR), and Mann-Whitney test.

## Results

The sample cases of 80 children with asthma had a mean (SD) age of 71.15 (33.52) months and an M:F ratio of 1.96:1. AR, AC, and AD were associated in 76 (95.0%), 33 (41.3%), and 22 (27.5%) cases, respectively. The mean (SD) absolute eosinophil count was 576.1 (427.5) per cmm. Total serum IgE >150IU/ml was found in 64 (82.5%) children. The mean (SD) total IgE was 800.9 (883.2) IU/ml. Seasonal variation and diurnal variations were found in 34 (42.5%) and 79 (98.8%) cases. Indoor was the major location for the symptoms. Important triggering agents for wheezing reported by parents were found out to be "sleeping/lying in bed'" and "exercise" in 23 (31.9%) and 7 (9.7%), respectively (Table 1).

**Table 1:** Baseline characteristics of children with asthma (n=80) Data presented as n (%). *Mean (SD). **Median (IQR). IgE, immunoglobulin E; M, male; F, female

Variable	P-value
Sex (M:F)	1.96:1
Age^*^ (month)	71.15 (33.52)
Residence	
Rural	26 (32.5)
Urban	54 (67.5)
BMI^*^ (kg/m^2^)	17.4 (3.7)
Associated features	
Allergic rhinitis	76 (95.0)
Allergic conjunctivitis	33 (41.3)
Allergic dermatitis	22 (27.5)
Eosinophil count^*^ (%)	5.26 (3.11)
Absolute eosinophil count* (per cmm)	576.1 (427.5)
Total IgE^*^ (IU/ml)	800.9 (883.2)
Total IgE^**^ (IU/ml)	435.5 (245.8, 1044.8)
IgE >150 IU/ml	64 (82.5)
Diurnal variation present	79 (98.8)
Seasonal variation present	34 (42.5)
Symptom location	
Indoor	43 (53.8)
Outdoor	23 (28.7)
Both (indoor and outdoor)	9 (11.3)
Triggers	
Walking in a garden	1 (1.4)
Sleeping/lying in bed	23 (31.9)
Exposure to animals/pets	1 (1.4)
Dust/dusty atmosphere	5 (6.9)
Consumption of particular foods	1 (1.4)
Physical exertion/exercise	7 (9.7)

**Figure 1:**
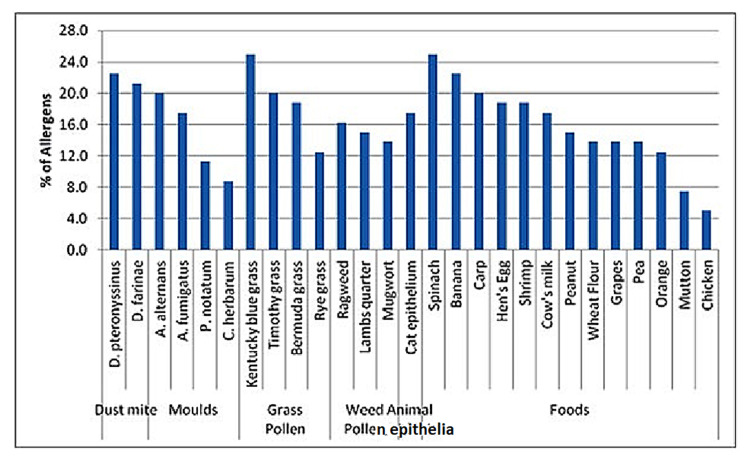
Prevalence of different aeroallergen and food allergen sensitization in childhood asthma

Figure 1 depicts the allergic sensitization in SPT in children with asthma. The most common sensitive aeroallergens were Kentucky bluegrass (25%), Dermatophagoides pteronyssinus (22.5%), Dermatophagoides farinae (21.3%), Timothy grass, and Alternaria alternans (20% each). The most common sensitive food allergens were spinach (25%), banana (22.5%), carp (20%), shrimp and hen’s egg (18.8% each), and cow’s milk (17.5%). 

**Figure 2:**
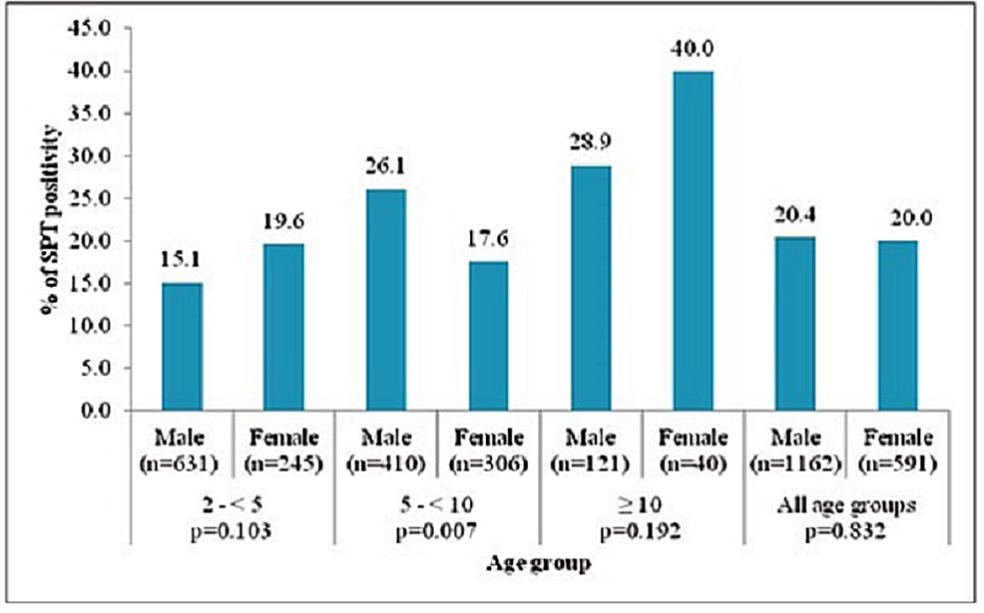
Age and sex distribution of SPT positivity against different allergen groups. SPT, skin prick test

Table 2 and Figure 2 compare the age and sex distribution of overall SPT positivity. Twenty-seven reagents were tested and out of 1753 skin pricks, 355 (20.25%) tests were positive. The increase in age is significantly associated with the increase in SPT positivity (P = 0.000). Overall, there is no significant association between SPT positivity and gender.

**Table 2:** Age and sex distribution of SPT positivity against different allergen groups Data presented as n (%). SPT, skin prick test

Factors	Total	SPT positivity	P-value
Age (year)			
2-4	876	143 (16.3%)	0.000
5-9	716	161 (22.5%)	
≥10	161	51 (31.7%)	
Sex			
Male	1162	237 (20.4)	0.832
Female	591	118 (20.0)	

Table 3 shows the demographic characteristics and associated features of asthmatic children with different allergen sensitization. Dust mite and mold sensitization are found significantly more in older children compared to younger children (P = 0.045 and 0.026, respectively). There is no significant association of sensitivity of different allergen with gender, habitat, and BMI. Association of AR, AC, and AD are similar in different allergen sensitized groups. There is no significant difference with respect to eosinophil count, total serum IgE, seasonal and diurnal variation between different groups of allergen sensitization.

**Table 3:** Demographic characteristics and associated features of asthmatic children with different groups of allergen sensitization Data presented as n (%). *Chi-square test. **Mean (SD). ***Mann-Whitney test. AR, allergic rhinitis; AC, allergic conjunctivitis; AD, atopic dermatitis; IgE, immunoglobulin E; AEC, absolute eosinophil count; SPT, skin prick test

Variable	Characteristics/sensitization	Dust mites (+ve 22, -ve 58)	Mold (+ve 36, -ve 44)	Grass pollen (+ve 43, -ve 37)	Weed pollen (+ve 27, -ve 53 )	Foods (+ve 65, -ve 15)
Sex	Male (n=53)	15 (28.3)	22 (41.5)	32 (60.4)	18 (34.0)	43 (81.1)
	Female (n=27)	7 (25.9)	14 (51.9)	11 (40.7)	9 (33.3)	22 (81.5)
	P-value^*^	0.822	0.379	0.096	0.955	0.970
Age^**^ (year)	2-4 (n=39)	6 (15.4)	12 (30.8)	19 (48.7)	11 (28.2)	32 (82.1)
	5-9 (n=33)	12 (36.4)	18 (54.5)	21 (63.6)	12 (36.4)	25 (75.8)
	≥10 (n=8)	4 (50.0)	6 (75.0)	3 (37.5)	4 (50.0)	8 (100)
	P-value^*^	0.045	0.026	0.280	0.453	0.284
Habitat	Rural	7 (26.9)	12 (46.2)	17 (65.4)	12 (46.2)	23 (88.5)
	Urban	15 (27.8)	24 (44.4)	26 (48.1)	15 (27.8)	42 (77.8)
	P-value^*^	0.936	0.886	0.148	0.104	0.252
BMI^**^ (kg/m^2^)	SPT +ve	18.2 (4.2)	18.2 (4.3)	17.1 (3.2)	17.4 (3.3)	17.4 (3.7)
	SPT -ve	17.1 (3.5)	16.9 (3.0)	17.8 (4.2)	17.4 (3.9)	17.5 (3.7)
	P-value^***^	0.301	0.230	0.647	0.768	0.921
AR (n=76)	SPT +ve	21 (95.5)	33 (91.7)	41 (95.3)	25 (92.6)	61 (93.8)
	SPT -ve	55 (94.8)	43 (97.7)	35 (94.6)	51 (96.2)	15 (100.0)
	P-value^*^	0.909	0.216	0.877	0.481	0.324
AC (n=33)	SPT +ve	11 (50.0)	15 (41.7)	16 (37.2)	10 (37.0)	25 (38.5)
	SPT -ve	22 (37.9)	18 (40.9)	17 (45.9)	23 (43.4)	8 (53.3)
	P-value^*^	0.328	0.945	0.429	0.585	0.292
AD (n=22)	SPT +ve	8 (36.4)	7 (19.4)	15 (34.9)	6 (22.2)	18 (27.7)
	SPT -ve	14 (24.1)	15 (34.1)	7 (18.9)	16 (30.2)	4 (26.7)
	P-value^*^	0.274	0.144	0.111	0.451	0.936
Eosinophil count^**^ (%)	SPT +ve	5.8 (2.9)	5.4 (3.0)	4.9 (2.2)	4.8 (2.7)	5.2 (3.0)
	SPT -ve	5.1 (3.2)	5.2 (3.3)	5.8 (3.9)	5.6 (3.3)	5.4 (3.8)
	P-value^***^	0.208	0.621	0.711	0.419	0.789
AEC^**^ (per cmm)	SPT +ve	572.09 (272.4)	534.7 (287.2)	517.8 (250.1)	510.7 (313.8)	572.2 (440.5)
	SPT -ve	577.7 (475.3)	610.0 (515.8)	643.1 (565.1)	609.4 (474.4)	593.0 (379.6)
	P-value^***^	0.265	0.942	0.696	0.545	0.902
Total IgE^**^ (IU/ml)	SPT +ve	1118.9 (1096.6)	902.4 (1014.9)	799.9 (874.3)	727.0 (836.1)	759.3 (812.8)
	SPT -ve	680.3 (764.3)	717.9 (760.9)	802.1 (905.5)	838.6 (911.7)	981.3 (1157.0)
	P-value^***^	0.071	0.820	0.559	0.915	0.786

Table 4 shows the demographic characteristics and associated features of asthmatic children with different food allergen sensitization. Cow's milk allergy is seen more in females compared to males (37% vs 7.5%, P = 0.001). A higher prevalence of food allergen sensitization is found with increasing age; statistically significant with cow's milk, peanut, meat, and seafood group (P = 0.037, 0.009, 0.020, respectively). The association of AR, AC, and AD is similar in different groups of food allergen sensitization. There is no significant difference with respect to eosinophil count, total serum IgE, seasonal and diurnal variation between different groups of food allergen sensitization (Table 4).

**Table 4:** Demographic characteristics and associated features of asthmatic children with different groups of food allergen sensitization Data presented as n(%). *Chi-square test. **Mean (SD). ***Mann-Whitney test. AR, allergic rhinitis; AD, atopic dermatitis; IgE, immunoglobulin E; AEC, absolute eosinophil count; SPT, skin prick test

Variable	Characteristics/ sensitization	Cow's milk (+ve 14, -ve 66)	Hen's egg (+ve 15, -ve 65)	Peanut (+ve 12, -ve 68)	Wheat flour (+ve 11, -ve 69)	Meat and seafood (+ve 34, -ve 46)	Fruits and veg. (+ve 42, -ve 38)
Sex	Male (n=53)	4 (7.5)	11 (20.8)	7 (13.2)	7 (13.2)	26 (49.1)	26 (49.1)
	Female (n=27)	10 (37.0)	4 (14.8)	5 (18.5)	4 (14.8)	8 (29.6)	16 (59.3)
	P-value^*^	0.001	0.520	0.529	0.844	0.096	0.388
Age (year)	2-4 (n=39)	5 (12.8)	5 (12.8)	3 (7.7)	6 (15.4)	21 (53.8)	21 (53.8)
	5-9 (n=33)	5 (15.2)	7 (21.2)	5 (15.2)	4 (12.1)	8 (24.2)	17 (51.5)
	≥10 (n=8)	4 (50.0)	3 (37.5)	4 (50.0)	1 (12.5)	5 (62.5)	4 (50.0)
	P-value^*^	0.037	0.237	0.009	0.917	0.020	0.970
Habitat	Rural (n=26)	5 (19.2)	4 (15.4)	6 (23.1)	3 (11.5)	12 (46.2)	15 (57.7)
	Urban (n=54)	9 (16.7)	11 (20.4)	6 (11.1)	8 (14.8)	22 (40.7)	27 (50.0)
	P-value^*^	0.777	0.593	0.160	0.690	0.646	0.519
BMI^**^ (kg/m^2 ^)	SPT +ve	18.4 (3.8)	18.2 (3.2)	18.4 (3.8)	18.1 (4.0)	17.6 (3.8)	17.1 (3.3)
	SPT -ve	17.2 (3.7)	17.3 (3.8)	17.2 (3.7)	17.3 (3.7)	17.3 (3.7)	17.8 (4.1)
	P-value^***^	0.186	0.147	0.241	0.463	0.665	0.600
AR (n=76)	SPT +ve	13 (92.9)	14 (93.3)	11 (91.7)	10 (90.9)	33 (97.1)	40 (95.2)
	SPT -ve	63 (95.5)	62 (95.4%)	65 (95.6)	66 (95.7)	43 (93.5)	36 (94.7)
	P-value^*^	0.685	0.742	0.566	0.503	0.468	0.918
AC (n=33)	SPT +ve	7 (50.0)	5 (33.3)	7 (58.3)	3 (27.3)	12 (35.3)	13 (31.0)
	SPT -ve	26 (39.4)	28 (43.1)	26 (38.2)	30 (43.5)	21 (45.7)	20 (52.6)
	P-value^*^	0.464	0.490	0.831	0.311	0.352	0.049
AD (n=22)	SPT +ve	2 (14.3)	7 (46.7)	2 (16.7)	4 (36.4)	11 (32.4)	13 (31.0)
	SPT -ve	20 (30.3)	15 (23.1)	20 (29.4)	18 (26.1)	11 (23.9)	9 (23.7)
	P-value^*^	0.223	0.065	0.362	0.478	0.403	0.467
Eosinophil count^**^ (%)	SPT +ve	6.0 (4.2)	5.6 (3.4)	4.5 (1.3)	4.1 (1.4)	5.4 (2.7)	4.9 (2.4)
	SPT -ve	5.1 (2.9)	5.2 (3.1)	5.4 (3.3)	5.4 (3.3)	5.2 (3.4)	5.8 (3.8)
	P-value^***^	0.626	0.955	0.668	0.219	0.301	0.504
AEC^**^ (per cmm)	SPT +ve	679.1 (753.9)	563.1 (351.7)	460.3 (110.0)	446.4 (183.5)	570.6 (308.5)	500.4 (271.8)
	SPT -ve	554.2 (325.1)	579.1 (445.5)	596.5 (459.1)	596.8 (451.9)	580.2 (501.1)	659.8 (542.8)
	P-value^***^	0.990	0.853	0.415	0.288	0.471	0.365
Total IgE^**^ (IU/ml)	SPT +ve	949.9 (822.2)	739.4 (1016.1)	825.8 (661.3)	263.9 (240.5)	800.1 (814.4)	705.3 (816.8)
	SPT -ve	769.3 (898.3)	815.1 (857.8)	796.5 (920.7)	886.5 (918.5)	801.5 (939.7)	906.6 (950.9)
	P-value^***^	0.175	0.398	0.377	0.010	0.903	0.223

## Discussion

In the present study, children with asthma were assessed for associated atopy, IgE assay, and skin prick tests to identify IgE-mediated allergen sensitization. History taking and documentation of temporal association to identify allergen were difficult to predict an allergen responsible for wheezing attacks in these children.

The majority (n=56, 80%) cases showed IgE level >150IU/ml, and the mean total IgE was 800.9 ± 883.2IU/ml. This is similar to the study by Rathoria et al. who reported the mean IgE levels in childhood asthma to be 881.81IU/ml [13]. This is consistent with previous reports suggesting serum IgE be characteristics of most allergic diseases including asthma [14-16]. Further, raised levels are neither sensitive nor specific for allergy diagnosis and documented in different conditions like a parasitic infestation, immunodeficiency disorders, Epstein-Barr virus infection, rheumatoid arthritis [17,18]. Other atopic manifestations like AR, AC, and AD were associated with asthma in the present study similar to the previous report. These findings suggest an association of atopy to wheezing in these children [19].

Several studies on SPT using different reagents have been done at different geographical locations with healthy children as well as those with wheezing and allergic diseases. In a study of SPT on 102 children with wheezing or allergic diseases, 61.8%(63/102) had sensitization to one or more allergen [20]. Skin prick tests were positive in 14% and 23% in wheezing infants at six months and 12 months, respectively [21]. We found 20.25% SPT positivity in our cohort of asthmatic children aged two to 14 years. Another study had reported positivity to at least one allergen in 13.5% of children [22]. However few studies from India reported higher SPT positivity; Northern India reported 55.6%( 100/180) in children above five years and a study from Mumbai children with allergic rhinitis, wheezing and eczema showed an SPT positivity of 53.2%.

In the present study, we found the prevalence of SPT positivity is increased significantly with increasing age (P = 0.001). These findings might be attributable to the natural history of atopic disorders (atopic march) where exposure to outdoor allergens occurring in later childhood. This is similar to the observation by Al-Zayadneh et al. who reported SPT positivity increasing significantly with age [19,23]. Aeroallergen sensitization in wheezing children (n=100) was studied by Pendino et al. and found sensitization to dust mite, pollen, and mold to be 58%, 13%, and 8% respectively [24]. A study from Northern India showed 36.7% sensitization to housefly antigen and 7.8% to house dust mite [8]. Our findings were lower (27.5%) in the dust mite group but higher in the pollen (53.7%) and mold (45%) group. This might be due to the inclusion of younger children (six months to 10 years) in the study by Pendino et al. supporting the fact that indoor allergens exposure occurs earlier in childhood [24,25].

The sensitivity of patients to different groups of allergens (pollens, molds, mites, animal dander, foods) is different in different studies. Breborowicz et al. reported most frequent allergy was timothy, house dust mite, birch, mugwort, cat dander and dog dander [22]. We found timothy, dust mite, and Kentucky bluegrass sensitization in about 20% each with a lower prevalence of mugwort (13.82%) and cat epithelia (17.5%) sensitization in our study. Olive tree pollen, cat fur allergen, and house dust mites were the most common inhaled allergens in children from Al-Karak as reported by Al-Zayadneh et al. [19]. Raj et al. reported an SPT positivity to rice grain dust and house dust mite to be 31.1% and 7.8%, respectively [8]. There is a paucity of data from different parts of India regarding the allergen prevalence and sensitization in childhood asthma and further studies using different allergens in other parts of the country may be useful.

We found a similar positive rate of SPT for mite as compared to Luo et al. (27.5% vs 24.2%) [20]. D. pteronyssinus and D. farinae are the two predominant mites tested in the present study with a positivity rate of 22.5% and 21.3%, respectively. Doshi and Tripathi and Al-Zayadneh et al. reported the most predominant mite to be D. pteronyssinus similar to our study [19,23]. However, Luo et al. reported significantly high positivity in SPT for D. farinae as compared to D. pteronyssinus (50.0% vs. 14.7%; P<0.05) [20].

We found 17.5% SPT positivity to cow's milk in children with asthma which is much higher than the reported prevalence of cow's milk hypersensitivity (0.59%) in a study of 1015 healthy infants aged eight months to 18 months by Kucukosmanoglu et al. [26]. In the study of Kucukosmanoglu et al., the prevalence of egg sensitization based on skin prick test results has been found to be 1.87% among Turkish healthy infants in Istanbul, which is much lesser than the SPT positivity to hen's egg (18.8%) in our children [27]. This suggests a higher prevalence of cow's milk allergy and egg allergy in asthmatic children as compared to healthy children. 

The key for allergy diagnosis lies in detailed clinical history including periodicity of symptoms (seasonal/perennial, diurnal), temporal association of allergen and symptoms, aggravating and relieving factors, occupational exposure, pet, and insects exposure. The important history of triggers reported by parents was "sleeping/lying in bed," "exercise," and "dust exposure" in 31.9%, 9.7%, and 6.9%, respectively. Most of them (98.8%) reported diurnal variations and 42.5% reported seasonal variations. The trigger factors, seasonal and diurnal variations were not predictive of the allergen responsible for the attacks, which might be due to the overlapping of multiple allergens causing symptoms in a particular child.

Limitation

The study was conducted in a single center and few selected standardized reagents were tested as per the availability. As the allergy test was restricted to SPT, the results can't exclude non-IgE mediated allergy. However, SPT forms a simple, cost and time-effective tool for the detection of allergic sensitization in childhood asthma specific for a geographical location. It provides a scientific basis for guiding clinicians for the initiation of avoidance of aeroallergens and food restriction.

## Conclusions

Childhood asthma is commonly associated with eosinophilia, increased serum IgE and a significant rate of SPT positivity; suggestive of atopy. Increasing age is significantly associated with increasing SPT positivity. Cow's milk allergy is seen more in females compared to males. The most common sensitive aeroallergens were Kentucky bluegrass and Dermatophagoides pteronyssinus, whereas spinach and banana were the most common food allergen.
